# Error-Free Bypass of 7,8-dihydro-8-oxo-2′-deoxyguanosineby DNA Polymerase of *Pseudomonas aeruginosa* Phage PaP1

**DOI:** 10.3390/genes8010018

**Published:** 2017-01-07

**Authors:** Shiling Gu, Qizhen Xue, Qin Liu, Mei Xiong, Wanneng Wang, Huidong Zhang

**Affiliations:** 1 College of Pharmacy and Bioengineering, Chongqing University of Technology, No. 29 Hongguang Street, Banan District, Chongqing 400054, China; shilinggu@foxmail.com (S.G.); liuqin@2014.cqut.edu.cn (Q.L.); 2 Public Health Laboratory Sciences and Toxicology, West China School of Public Health, Sichuan University, No. 17 People’s South Road, Chengdu 610041, China; xueqzhen@163.com (Q.X.); xhmei2016@126.com (M.X.)

**Keywords:** *P. aeruginosa* phage PaP1, DNA polymerase, 8-oxoG, steady-state kinetics, pre-steady-state kinetics, nucleotide incorporation

## Abstract

As one of the most common forms of oxidative DNA damage, 7,8-dihydro-8-oxo-2′-deoxyguanosine (8-oxoG) generally leads to G:C to T:A mutagenesis. To study DNA replication encountering 8-oxoG by the sole DNA polymerase (Gp90) of *Pseudomonas* *aeruginosa* phage PaP1, we performed steady-state and pre-steady-state kinetic analyses of nucleotide incorporation opposite 8-oxoG by Gp90 D234A that lacks exonuclease activities on ssDNA and dsDNA substrates. Gp90 D234A could bypass 8-oxoG in an error-free manner, preferentially incorporate dCTP opposite 8-oxoG, and yield similar misincorporation frequency to unmodified G. Gp90 D234A could extend beyond C:8-oxoG or A:8-oxoG base pairs with the same efficiency. dCTP incorporation opposite G and dCTP or dATP incorporation opposite 8-oxoG showed fast burst phases. The burst of incorporation efficiency (*k*_pol_/*K*_d_,_dNTP_) is decreased as dCTP:G > dCTP:8-oxoG > dATP:8-oxoG. The presence of 8-oxoG in DNA does not affect its binding to Gp90 D234A in a binary complex but it does affect it in a ternary complex with dNTP and Mg^2+^, and dATP misincorporation opposite 8-oxoG further weakens the binding of Gp90 D234A to DNA. This study reveals Gp90 D234A can bypass 8-oxoG in an error-free manner, providing further understanding in DNA replication encountering oxidation lesion for *P.aeruginosa* phage PaP1.

## 1. Introduction

Accurate synthesis of DNA is of great importance for genomic integrity in all forms of life. DNA replication is generally performed by DNA polymerases with high fidelity. Accurate DNA replication is under constant threat, which are formed within the genome [1]. DNA damage incurred by a multitude of factors constitutes an unavoidable challenge for the replication machinery [2]. Reactive oxygen species are a major source of DNA damage. One of the most common lesions induced by oxidative stress is 7,8-dihydro-8-oxo-2′-deoxyguanosine (8-oxoG) [3], which is representative of nucleoside damage and shows genotoxicity [4]. The deleterious effects of 8-oxoG on DNA replication can be attributed to its dual-coding potential that leads to G:C to T:A transversions [5].

Lesion tolerance is achieved, in part, by special translesion synthesis (TLS) polymerases, which are able to bypass lesions during DNA replication [6]. Compared with replicative DNA polymerases, TLS polymerases have relatively larger active sites, allowing them to accommodate mismatched base pairs and bulky DNA lesions at the cost of a lower fidelity [7,8]. Most DNA polymerase nucleotides incorporate opposite template 8-oxoG lesions with reduced efficiency and accuracy [9]. Error-free DNA synthesis involves 8-oxoG adopting an anti-conformation to the base pair with cytosine, whereas a mutagenic bypass involves 8-oxoG adopting a syn-conformation to the base pair with adenine [10].

Human DNA polymerase α, human DNA polymerase η, and *Bacillus stearothermophilus* DNA Polymerase I are capable of bypassing 8-oxoG in a mostly error-free manner [11]. The sole Y-family DNA polymerase Dpo4 in *Sulfolobus solfataricus*, can efficiently and reliably incorporate dCTP opposite 8-oxoG and extend from an 8-oxoG:C base pair with a mechanism similar to the bypass of undamaged DNA [12]. In crystal structures, Arg-332 in Dpo4 stabilizes the anti-conformation of the 8-oxoG template base, which results in increased efficiency for dCTP insertion and less favorable formation of a Hoogsteen pair between 8-oxoG and dATP [13].

Human DNA polymerase ι is error-prone in 8-oxoG bypass with low fidelity [14]. Human DNA polymerase β has similar efficiencies for dCTP and dATP insertion opposite 8-oxoG [15]. Human DNA polymerase κ bypasses 8-oxoG in an error-prone manner by mainly inserting dATP. Crystal structures of hPol κ ternary complex reveal nonproductive alignments of incoming nucleotides (dGTP or dATP) with 8-oxoG. The interactions between the N-clasp and finger domains of hPol κ stabilize the *syn* orientation of 8-oxoG that contributes to error-prone dATP incorporation. Mutation of Leu-508 into lysine at the little finger domain of hPol κ modulates the insertion of dCTP opposite 8-oxoG, leading to more accurate bypass [16].

*Pseudomonas aeruginosa* is difficult to treat because of drug resistance. PaP1, as a lytic phage of *P. aeruginosa*, is a potential alternative to treat *P. aeruginosa* infections. Recently, our group has identified that DNA polymerase (Gp90) in *P. aerugiosa* phage PaP1 is an A-family DNA polymerase containing ssDNA and dsDNA exonuclease activities [17]. As the sole DNA polymerase in PaP1 [18], Gp90 has a significant role in DNA synthesis in PaP1 propagation. Studies on DNA replication by Gp90 will contribute to our understanding of PaP1 propagation in hosts infected with *P. aeruginosa*. As one of the most common oxidation lesions, 8-oxoG may affect DNA replication by Gp90. We performed steady-state and pre-steady-state kinetic analyses of nucleotide incorporation opposite, or beyond, 8-oxoG using Gp90 to understand how PaP1 bypasses 8-oxoG.

During nucleotide incorporation, the nucleotide binding step, conformational change, and chemistry steps are three important elementary steps that directly determine the fidelity of 8-oxoG bypass [19]. Steady-state kinetic analysis of nucleotide incorporation can provide information on enzyme specificity and efficiency, but cannot determine these elementary steps. With the pre-steady-state kinetic method, these elementary steps are directly examined in the first turnover, eliminating the effect of the subsequent slow dissociation of polymerase from the DNA [20]. In this study, we performed steady-state and pre-steady-state kinetic analyses of dNTP incorporation opposite, or beyond, 8-oxoG and determined that Gp90 can ensure error-free bypass of 8-oxoG, indicating that PaP1 can tolerate oxidation lesions during its propagation.

## 2. Materials and Methods

### 2.1. Materials

Mutagenesis was performed using a QuikChange site-directed mutagenesis kit (Stratagene, La Jolla, CA, USA). Oligonucleotides were synthesized by Midland Certified Reagent Co (Midland, TX, USA). All unlabeled dNTPs and T4 polynucleotide kinases were obtained from Amersham Bio-sciences (Piscataway, NJ, USA). Ni-NTA mini-spin columns were purchased from GE Healthcare (Pittsburgh, PA, USA). [γ-^32^P] ATP was obtained from PerkinElmer Life Sciences (Boston, MA, USA). Bio-spin columns were obtained from Bio-Rad (Hercules, CA, USA). Phage PaP1 was propagated and extracted, and its genomic DNA was extracted and purified as described previously [21,22]. Other commercially available reagents were of the highest quality.

### 2.2. Construction, Expression, and Purification of Gp90 Mutants

Glu-60, Asp-137 and Asp-234 were predicted as the potential exonuclease active residues of Gp90 by alignment of the sequences of Gp90, T7 DNA polymerase, and *Escherichia coli* polymerase I [17]. These residues in Gp90 were then mutated to alanine using a QuikChange site-directed mutagenesis kit. Three Gp90 mutants (Gp90 E60A, Gp90 D234A, and Gp90 E60A D137A D234A) were prepared. The primers used for mutagenesis were listed below:

E60A: sense, 5′-GTAGTAGCCGCCCACGGCGGTAACATTCTGGCGTTCTAC-3′;
  antisense, 5′-GCC GTGGGCGGCTACTACGACACAGTGATGACTGTAGCT-3′.
D137A: sense, 5′-ATTAACTTCGCCCTTATGTCGATGAAGCTTGTGGAAGATATG-3′;
  antisense, 5′-CGACATAAGGGCGAAGTTAATCATGTTGTGAGCCACTACGCG-3′.
D234A: sense, 5′-TGTATCTATGCCGTAAAGGCGAACACCGCTGTATGGCACTGG-3′;
  antisense, 5′-CGCCTTTACGGCATAGATACAGTAGTAAAGCATATCGGCTGC-3′.

The DNA sequences were confirmed by sequence analysis prior to bacterial expression. Wild-type and three mutants were expressed in *E. coli* A307 (DE3) cells, followed by purification through a HisTrap^TM^ FF column (5 mL; GE Healthcare) as described previously [17].

### 2.3. Examination of Exonuclease Activities of Gp90 Mutants

The ssDNA or dsDNA exonuclease activities were determined by mixing 10 nM each of DNA polymerase with 20 nM ^32^P-labeled 27-mer ssDNA or ^32^P-labeled 27-mer/62-mer primer/template dsDNA, respectively, in a buffer containing 40 mM Tris-HCl (pH 7.5), 30 mM Mg^2+^ and 10 mM DTT at 37 °C. After 0.5, 1, 2, or 5 min, reactions were terminated with a quench solution containing 20 mM EDTA, 95% formamide (v/v), bromphenol blue, and xylene cyanol. The samples were then separated on a 20% polyacrylamide (w/v)/7 M urea gel. Products were visualized and quantified using a phosphorimaging screen and Quantity One^TM^ software (Bio-Rad, Hercules, CA, USA) [23].

### 2.4. Primer Extension by Gp90 Mutants Using All Four dNTPs

A ^32^P-labeled 27-mer primer, annealed to 62-mer template oligonucleotide, was extended in the presence of all four dNTPs in a buffer containing 40 mM Tris-HCl (pH 7.5), 30 mM Mg^2+^, 10 mM DTT, and 50 mM potassium glutamate at 37 °C. The reactions were initiated by mixing 20 nM DNA substrates with 10 nM Gp90 or Gp90 mutants and 350 μM each of dNTP for 0.5, 1, 2, or 5 min. Reactions were terminated by a quench solution containing 20 mM EDTA, 95% formamide (v/v), bromphenol blue, and xylene cyanol. The samples were then separated on a 20% polyacrylamide (w/v)/7 M urea gel. Products were visualized and quantified using a phosphorimaging screen and Quantity One^TM^ software. Primer extension beyond 8-oxoG were performed similarly by mixing 20 nM DNA substrates containing 8-oxoG with 10 nM Gp90 or Gp90 D234A and 350 μM each of dNTP and reacted for 0.5, 1, 2, or 5 min.

### 2.5. Steady-State Kinetics Analysis of Single-Base Incorporation and Next-Base Extension

Steady-state kinetic analysis of single-base incorporation and next-base extension by Gp90 D234A were performed using ^32^P-labeled 27-mer/62-mer and ^32^P-labeled 28-mer/62-mer dsDNA substrates, respectively (Table 1). The molar ratio of Gp90 D234A to DNA substrate was <0.10. The concentration of Gp90 D234A and reaction time were adjusted to control the extension of the primer <0.2 [24]. All reactions were performed in buffer containing 40 mM Tris-HCl (pH 7.5), 30 mM Mg^2+^, 10 mM DTT, and 50 mM potassium glutamate. Reactions products were analyzed by gel electrophoresis, visualized using phosphorimaging and quantified by Quantity One^TM^ software. Graphs of product formation rates versus dNTP concentrations were fit by nonlinear regression (hyperbolic fits) using GraphPad Prism Version 6.0 (San Diego, CA, USA) to determine *k*_cat_ and *K*_m_ values [25]. The misincorporation frequencies were calculated by dividing the misincorporation efficiency (*k*_cat_/*K*_m_) of incorrect dNTP by that of the correct dCTP.

### 2.6. Pre-Steady-State Kinetic Analysis

Rapid chemical quench experiments were performed using a model RQF-3 KinTek Quench Flow Apparatus (KinTek Corp, Austin, TX, USA) with 50 mM Tris-HCl (pH 7.5) buffer in the drive syringes [26]. Reactions were initiated by rapidly mixing 240 nM ^32^P-labeled 27-mer/62-mer primer/template dsDNA substrate and 160 nM Gp90 D234A mixtures with an equal volume of 2 mM dNTP and 60 mM Mg^2+^ complex, incubated for a varied time (0.005–60 s) at 37 °C, and then quenched with 0.6 M EDTA. Substrate and product DNA were separated by electrophoresis on a 20% polyacrylamide (w/v)/7 M urea gel. The products were then visualized using phosphorimaging and quantified using Quantity One^TM^ software. The product and time were fit to Equation (1), corresponding dNTP incorporation in the first binding phase and the subsequent steady-state phase:

*y* = *A* (1 − e*^k^*^p *t*^) + *k*_ss_*t* (1)

where *A* is the amount of active complex formed in the first binding phase, nM; *k*_p_ is the dNTP incorporation rate in the first binding phase, s^−1^; *k*_ss_ is the rate of steady-state dNTP incorporation, s^−1^; and *t* is time, s^−1^.

Nucleotide incorporation rates in the first binding phase could also be determined by rapidly mixing 400 nM Gp90 D234A and 200 nM DNA with an equal volume of different concentrations of dNTP and 60 mM Mg^2+^, and incubated for a varied time. The product amount and time were fit to Equation (2), corresponding dNTP incorporation only in the first binding phase:

*y* = *A* (1 − e^−*k*obs *t*^) (2)

where *A* is the amount of active complex formed in the first binding phase, nM; *k*_obs_ is the dNTP incorporation rate in the first binding phase (burst rate), s^−1^; and *t* is time, s^−1^.

Burst rates (*k*_obs_) and concentrations of dNTP were fit to hyperbolic Equation (3) to obtain *k*_pol_ and *K*_d,dNTP_ values:

*k*_obs_= *k*_pol_ [dNTP]/([dNTP]+ *K*_d,dNTP_) (3)

where *k*_pol_ is the maximal rate of dNTP incorporation, s^−1^; and *K*_d,dNTP_ is the equilibrium dissociation constant for dNTP in the burst phase, μM [27].

### 2.7. Biophysical Binding of Gp90 D234A to DNA Containing G or 8-oxoG

Surface plasmon resonance analysis was performed by using a Biacore-3000 instrument (Biacore, Uppsala, Sweden) [22]. An annealed DNA (600 RU) consisting of a primer (5′-Biotin-TTTGCTACAGAGTTATGGTGACGATACGTC_dd_-3′) and a template (5′-TGAATTCTAAT GTAGTATAGTAATCCGCTCTATCGGACGTATCGTCACCATAACTCTGTAGC-3′) was coupled to a streptavidin (SA) chip. The C_dd_ (double deoxycytosine) at the 3′-end of primercan stop DNA polymerization. A varying concentration of Gp90 or Gp90 D234A (20-600 nM) was flowed over the chip in a buffer containing 40 mM Tris-HCl (pH 7.5), 10 mM DTT, and 50 mM potassium glutamate at a flow rate of 10 µL/min at room temperature. In a control flow cell, biotin was used instead of the biotinylated DNA to compensate for background. The chip surface was regenerated by injection of 1 M NaCl solution at a flow rate of 100 μL/min. The binding signal was fitted to Equation (4) using a steady-state model provided by BIA evaluation 3.0.2 computational software (Biacore). The dissociation constants *K*_d_ were calculated using the steady-state average RU.

*Y* = *B* × RU_max_/(*B* + *K*_d_) (4)

where *Y* is the response signal corresponding to the binding, RU; *B* is the concentration of protein, nM; RU_max_ is the maximal binding amount, RU; and *K*_d_ is the dissociation constant, nM. All experiments were carried out three times, and standard errors were derived using Prism 6.0 software.

The similar binding assays were also performed in the presence of 30 mM MgCl_2_ and 1 mM dCTP or dATP. In the presence of Mg^2+^ and dNTP, the active site of DNA polymerase would locate at the 3′-end of primer where dNTP is paired or mispaired opposite G or 8-oxoG in the template strand. The binding affinities of the polymerase to DNA were determined by the same methods as described above.

## 3. Results

### 3.1. Examination of Exonuclease and Polymerase Activities of Gp90 Mutants

**Figure 1:**
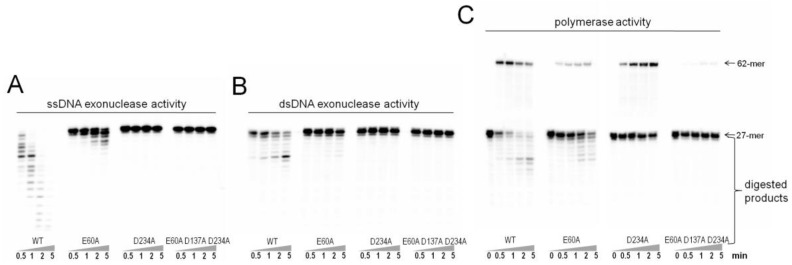
(**A**) ssDNA exonuclease assays. ssDNA exonuclease activities of Gp90 mutants were examined by mixing 10 nM each of DNA polymerase with 20 nM ^32^P-labeled 27-mer ssDNA in a buffer containing 40 mM Tris-HCl (pH 7.5), 30 mM Mg^2+^, and 10 mM DTT at 37 °C for 0.5, 1, 2, or 5 min. (**B**) dsDNA exonuclease assays. dsDNA exonuclease activities of Gp90 mutants were examined as described above, except for using ^32^P-labeled 27-mer/62-mer dsDNA substrate. (**C**) Polymerase activity assays. Polymerase activities of Gp90 mutants were examined by mixing 10 nM polymerase with 20 nM ^32^P-labeled 27-mer/62-mer dsDNA substrate and 350 μM each of dNTP in the same reaction buffer for 0.5, 1, 2, or 5 min. Representative data from multiple experiments are shown.

Glu-60, Asp-137, and Asp-234 were predicted as the potential exonuclease active residues of Gp90 by alignment of the sequences of Gp90, T7 DNA polymerase and *E. coli* polymerase I [17]. SsDNA exonuclease activities of Gp90 and its three mutants were tested using a ^32^P-labeled 27-mer ssDNA (Figure 1A). With increasing the reaction time, short ssDNA products were gradually produced by Gp90 and Gp90 E60A, demonstrating 3′–5′ ssDNA exonuclease activity. SsDNA exonuclease activity of Gp90 E60A was partially decreased compared with Gp90. Gp90 D234A and Gp90 E60A D137A D234A showed no obvious degraded ssDNA products, indicating that their ssDNA exonuclease activities have been eliminated.

DsDNA exonuclease activities were examined using ^32^P-labeled 27-mer/62-mer primer/template dsDNA substrate (Figure 1B). Compared with wild-type Gp90, Gp90 E60A exhibited partial dsDNA exonuclease activity. Short degraded products were gradually produced, demonstrating 3′–5′ dsDNA exonuclease activities. Gp90 D234A and Gp90 E60A D137A D234A were deficient in dsDNA exonuclease activity. Polymerase activities of Gp90 and its three mutants were also examined by full-length extension of a ^32^P-labeled 27-mer/62-mer dsDNA substrate in the presence of all four dNTPs. The 27-mer primer was readily extended to 62-mer by Gp90 and Gp90 D234A (Figure 1C). Gp90 E60A showed partial polymerase activity and Gp90 E60A D137A D234A almost lost its polymerase activity. Therefore, mutation of Asp-234 to Ala can efficiently abolish exonuclease activity, but can almost retain the polymerase activity. Gp90 D234A was then used asexonuclease-deficient in this work.

### 3.2. Primer Extension beyond 8-oxoG by Gp90 D234A Using All Four dNTPs

**Figure 2:**
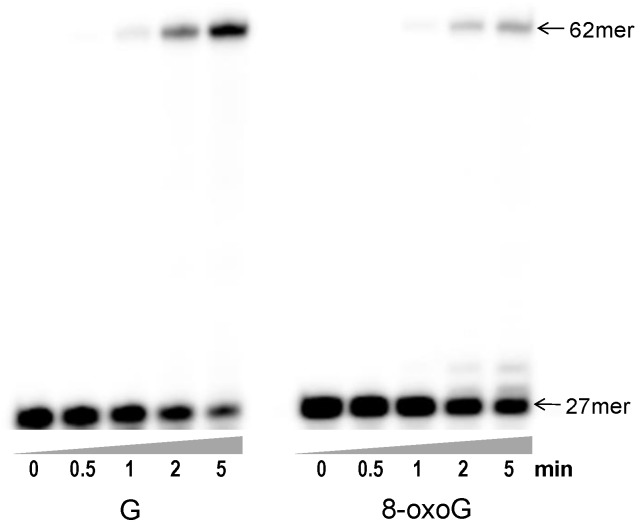
Extension of ^32^P-labeled primer beyond 8-oxoG by Gp90 D234A in the presence of all four dNTPs. Extension assays were performed by mixing 10 nM Gp90 D234A, 20 nM ^32^P-labeled 27-mer/62-mer dsDNA substrate, and 350 μM each of dNTP in a reaction buffer as described in Materials and Methods. Representative data from multiple experiments are shown.

Gp90 D234A readily extended 27-mer primer to 62-mer on unmodified template G (Figure 2). No intermediate product bands were observed, similar to previous results that Gp90 was a highly processive DNA polymerase [17]. The extension beyond 8-oxoG was partially inhibited, as evidenced by the presence of more unextended primer and less full-length extended products. Some 28-mer and 29-mer products were also observed for 8-oxoG. Therefore, the presence of 8-oxoG in the template partially inhibited DNA polymerization.

### 3.3. Steady-State Kinetic Analysis of Single-Base Incorporation Opposite G or 8-oxoG by Gp90 D234A

The steady-state kinetic parameters (i.e., *k*_cat_ and *K*_m_) for each single dNTP incorporation opposite G or 8-oxoG by Gp90 D234A were measured (Table 2). dCTP was preferentially incorporated opposite G and the misincorporation frequencies of other dNTPs were in the range of 10^−4^ to 10^−5^. In detail, the *k*_cat_ values of all four dNTPs were similar, but the *K*_m_ values of three incorrect dNTPs were significantly increased compared with that of dCTP. For 8-oxoG, dCTP was still highly preferentially incorporated and the misincorporation frequencies of other dNTPs were in the range of 10^−4^ to 10^−5^. Notably, all of incorporation efficiencies opposite 8-oxoG were significantly reduced compared with those opposite G. The efficiency of dCTP incorporation opposite 8-oxoG was reduced by 700-fold compared with that of dCTP opposite G because of the increased *K*_m_ value, but unchanged *k*_cat_ value. dNTP misincorporation opposite 8-oxoG generally resulted in higher *K*_m_ and lower *k*_cat_ values compared with that of dCTP incorporation opposite 8-oxoG.

### 3.4. Steady-State Kinetic Analysis of Next-Base Extension beyond G or 8-oxoG by Gp90 D234A

Th steady-state kinetic parameters (i.e., *k*_cat_ and *K*_m_) for the next-base extension beyond G or 8-oxoG by Gp90 D234A (Table 3) were measured. The C or A at the 3′-end of primer was paired or mispaired with template G or 8-oxoG, respectively (Table 1). dGTP was incorporated opposite the next template base C. The incorporation efficiency was approximately 10-fold higher in extension beyond C:G (primer:template) than A:G because of the higher *k*_cat_, but unchanged *K*_m_ values. For template 8-oxoG, both C:8-oxoG and A:8-oxoG base pairs were similarly extended by Gp90 D234A, but the efficiencies were reduced 490-fold compared with the extension beyond the C:G base pair because of the increased *K*_m_ values. Gp90 D234A preferentially extended the C:G base pair rather than the A:G mispair, but extended beyond C:8-oxoG or A:8-oxoG base pairs with the same efficiency.

### 3.5. Pre-Steady-State Kinetic Analysis of Single dNTP Incorporation by Gp90 D234A

Generally, incorporation of a correct dNTP by most DNA polymerases shows a biphasic character (burst phase and linear steady-state phase) [23]. In the burst phase, dNTP is quickly incorporated opposite the template base during the first binding of polymerase to DNA (the first turnover); in the linear steady-state phase, polymerase is dissociated from DNA, then binds to DNA and incorporates dNTP, all of which are limited by the slow dissociation of polymerase from DNA. The presence of biphasic shapes indicates that dNTP incorporation is much faster than the subsequent dissociation of polymerase from DNA.

**Figure 3:**
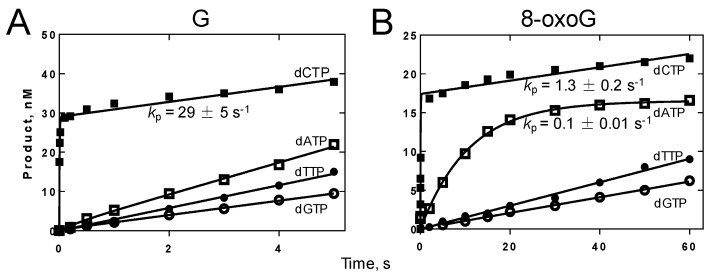
Pre-steady-state kinetic analysis of nucleotide incorporation by Gp90 D234A. Gp90 D234A (80 nM) was incubated with 100 nM ^32^P-labeled 27-mer/62-mer primer/template containing G (**A**) or 8-oxoG (**B**), 1 mM each individual dNTP, and 30 mM Mg^2+^ in a RQF-3 KinTek quench flow apparatus as described in Materials and Methods. Representative data from multiple experiments are shown.

Excess molar concentration of DNA, compared with polymerase, was used to determine the rates of dNTP incorporation opposite G or 8-oxoG by Gp90 D234A in burst and linear steady-state phases [19]. Among four dNTPs, dCTP was preferentially incorporated opposite G and showed a fast burst phase, indicating that dCTP incorporation was faster than the dissociation of Gp90 D234A from DNA (Figure 3A). For dCTP, dATP, or dTTP incorporation opposite G, the product and reaction time exhibited a linear steady-state phase, without a fast burst phase. dCTP or dATP was preferentially incorporated opposite 8-oxoG and exhibited a fast burst phase (Figure 3B). dGTP or dTTP incorporations opposite 8-oxoG showed a linear steady-state phase. Therefore, dCTP or dATP incorporation opposite 8-oxoG was faster than the dissociation of Gp90 D234A from DNA and dTTP or dGTP incorporation opposite 8-oxoG.

**Figure 4:**
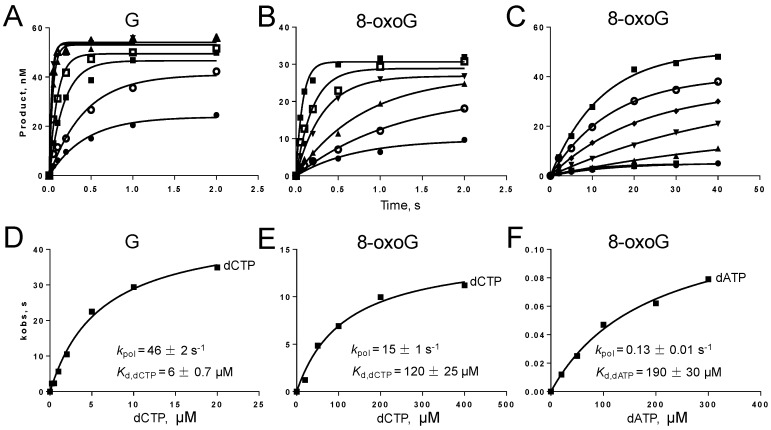
Pre-steady-state incorporation of a single dNTP opposite G or 8-oxoG by Gp90 D234A. Gp90 D234A (200 nM) incubated with 100 nM ^32^P-labeled 27-mer/62-mer primer/template complexes was fast mixed with varying concentrations of dCTP to initiate reactions in a rapid quench-flow instrument. Plots of product concentrations versus time were fit to a single exponential equation to obtain *k*_obs_ at every dCTP concentration. Then, plots of burst rates (*k*_obs_) versus dCTP concentrations were fit to a hyperbolic equation to obtain *k*_pol_ and *K*_d_ values. **A** and **D**, incorporation of dCTP opposite G. **B**, and **E**, incorporation of dCTP opposite 8-oxoG. **C** and **F**, incorporations of dATP opposite 8-oxoG. Representative data from multiple experiments are shown.

dCTP incorporation opposite G and dCTP or dATP incorporation opposite 8-oxoG exhibited fast burst phases. Moreover, the maximal rates of nucleotide incorporation (*k*_pol_) and the apparent dissociation constants of dNTP from the Gp90-DNA-dNTP ternary complex (*K*_d,dNTP_) were estimated by fitting the burst rates against dNTP concentrations to Equation (3) (Figure 4). *k*_pol_ of dCTP incorporation opposite G was 46 s^−1^ and *K*_d,dCTP_ was 6 µM. For dCTP incorporation opposite 8-oxoG, *k*_pol_ was decreased by three-fold, *K*_d,dCTP_ was increased by 20-fold, and total efficiency (*k*_pol_/*K*_d,dCTP_) was reduced by 59-fold compared with dCTP incorporation opposite G. The efficiency of dATP incorporation opposite 8-oxoG was further reduced by 188-fold compared with dCTP incorporation opposite 8-oxoG mainly because of the 115-fold reduction in *k*_pol_.

### 3.6. Binding of Gp90 D234A to the PrimerTemplate Containing 8-oxoG

**Figure 5:**
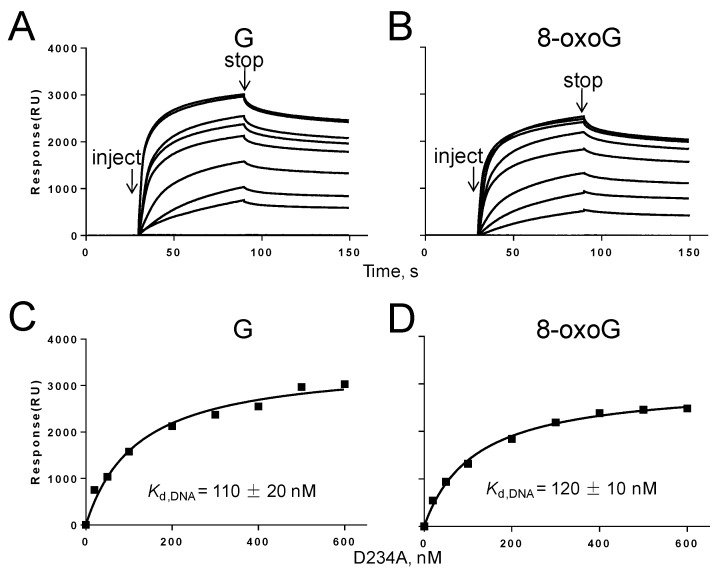
Biophysical binding of Gp90 D234A to DNA containing G or 8-oxoG in the absence of Mg^2+^ and dCTP. (**A**,**B**) Sensorgrams for binding of Gp90 D234A (20-600 nM) to DNA immobilized on the SA chip (300 RU) in buffer containing 40 mM Tris-HCl (pH 7.5), 10 mM DTT, and 50 mM potassium glutamate. (**C**,**D**) The binding affinities of Gp90 D234A to DNA were determined using the steady-state average response at each concentration of Gp90 D234A. The solid lines represent the theoretical curve calculated from the steady-state fit model (Biacore). Representative data from multiple experiments are shown.

The dissociation constants (*K*_d,DNA_) between DNA and DNA polymerase were determined by surface plasmon resonance to determine whether 8-oxoG affects the binding affinity of Gp90 D234A to DNA [28]. DNA containing G or 8-oxoG (300 RU) was immobilized on the SA chip and different concentrations of polymerase were flowed over the chip to measure the binding of polymerase to DNA. *K*_d,DNA_ was obtained by fitting the observed response signals against protein concentrations to Equation (4) using the steady-state model. In the absence of dNTP and Mg^2+^, DNA polymerase randomly bound to DNA to form a binary complex. The binding of polymerase to DNA containing G or 8-oxoG exhibited a similar *K*_d_ of 108 and 116 nM, respectively (Figure 5), indicating that 8-oxoG does not affect the binding affinity of Gp90 D234A to DNA.

**Figure 6:**
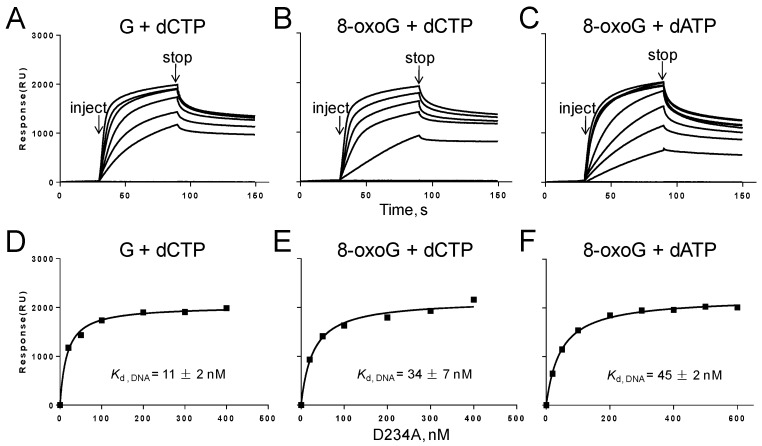
Biophysical binding of Gp90 D234A to DNA containing G or 8-oxoG in the presence of Mg^2+^ and dNTP. Scheme for measuring the interaction of Gp90 D234A with DNA that was prepared and immobilized onto the SA chip (300 RU). (**A**–**C**)Sensorgrams for the binding of Gp90 D234A (20-600 nM) to DNA immobilized on the SA chip (300 RU) in the presence of dCTP or dATP and Mg^2+^ in a buffer containing 40 mM Tris-HCl (pH 7.5), 10 mM DTT, and 50 mM potassium glutamate. (**D**–**F**)The binding affinities of Gp90 D234A to DNA were determined using the steady-state average response at each concentration of Gp90 D234A. The solid lines represent the theoretical curve calculated from the steady-state fit model (Biacore). Representative data from multiple experiments are shown.

In the presence of dNTP and Mg^2+^, DNA polymerase, DNA, and dNTP formed a ternary complex, in which polymerase was preferentially positioned at the 3′-end of the primer strand. The *K*_d,DNA_ values were significantly reduced compared with those without dNTP and Mg^2+^ (Figure 6). Therefore, the presence of dNTP and Mg^2+^ stabilized the binding of polymerase to DNA. Notably, *K*_d,DNA_ of 8-oxoG complex (34 nM) was three-fold higher than that of the G complex (11 nM) in the presence of dCTP and Mg^2+^, indicating that 8-oxoG in the template reduced the binding of Gp90 to DNA by three-fold. In the presence of dATP and Mg^2+^, *K*_d,DNA_ of 8-oxoG complex was further increased to 45 nM, showing that the presence of incorrect dATP further reduces the binding affinity of Gp90 D234A to DNA.

## 4. Discussion

Gp90 is the sole DNA polymerase responsible for DNA replication in PaP1 [18]. The exonuclease activity of Gp90 should be eliminated to analyze 8-oxoG bypass by Gp90 kinetically. In our previous results, Glu-60, Asp-137, and Asp-234 were predicted as the exonuclease active residues of Gp90 [29]. Gp90 D234A, in which Asp-234 was replaced with Ala, can efficiently abolish the ssDNA and dsDNA exonuclease activities while maintaining its polymerase activity (Figure 1). Thus, Asp-234 should be a crucial residue for exonuclease activity. This residue corresponds to Asp-501 in *E. coli* DNA polymerase I and Asp-174 in T7 DNA polymerase based on sequence alignment analysis. Furthermore, Gp90 D234A was used as exonuclease-deficient DNA polymerase for kinetic analysis in this work.

Steady-state kinetic analysis dNTP incorporation opposite G or 8-oxoG provides information on the efficiency and accuracy of DNA replication [30]. Among four dNTPs, dCTP is preferentially incorporated opposite either G or 8-oxoG, yielding misincorporation frequencies of 10^−4^ to 10^−5^ for G and 8-oxoG (Table 2). Gp90 D234A can accurately bypass 8-oxoG without obvious dATP misincorporation. However, the incorporation efficiencies opposite 8-oxoG are significantly reduced. These results further confirm that 8-oxoG partially inhibits primer extension compared with G in the presence of four dNTPs.

Similar to Gp90 D234A, *S. solfataricus* DNA polymerase Dpo4 [12] and human DNA polymerase η [2] can also accurately bypass 8-oxoG without obvious dATP misincorporation, although the incorporation efficiencies are partially reduced. By contrast, T7 DNA polymerase [31], yeast DNA polymerase η_core_ [24], human DNA polymerase ι [32] and human DNA polymerase β [15] lead to dCTP incorporation and obvious dATP misincorporation. Notably, human DNA polymerase κ [16] preferentially incorporates dATP opposite 8-oxoG and leads to G:C to T:A conversion.

Misincorporations opposite G inhibit next-base incorporation; whereas misincorporations opposite 8-oxoG do not affect next-base incorporation (Table 3). Gp90 D234A preferentially extends the C:G base pair rather than the A:G mispair but shows the same priority in extension beyond C:8-oxoG or A:8-oxoG.

For most DNA polymerases, incorporation of a correct dNTP shows a biphasic character; whereas misincorporation shows only a linear steady-state phase. Unexpectedly, dCTP or dATP incorporation opposite 8-oxoG by Gp90 D234A shows a biphasic character (Figure 3), although dATP incorporation efficiency (*k*_pol_/*K*_d,dATP_) is lower than that of dCTP. Similarly, human Y-family DNA polymerase κ also shows a fast burst phase for dATP incorporation opposite 8-oxoG [16]. T7 DNA polymerase exhibits a similar burst rate for dCTP or dATP incorporation opposite 8-oxoG [31]. However, for most DNA polymerases, dATP misincorporation opposite 8-oxoG shows only a linear steady-state phase without a fast burst phase [9].

The physical binding of Gp90 D234A to DNA containing G or 8-oxoG was measured. In the absence of dNTP or Mg^2+^, Gp90 D234A randomly binds to DNA to form a binary complex. The similar *K*_d,DNA_ values (Figure 5) show that 8-oxoG in the template does not affect the binding of Gp90 D234A to DNA. In the presence of dNTP and Mg^2+^, DNA polymerase is prone to bind at the 3′-end of the primer strand and form a ternary complex [22]. The lower *K*_d,DNA_ values of ternary complexes compared with binary complexes indicate that the presence of dNTP and Mg^2+^ stabilizes the binding of polymerase to DNA for G and 8-oxoG. Notably, the *K*_d,DNA_ value of 8-oxoG ternary complex was three-fold higher than that of G ternary complex in the presence of dCTP and Mg^2+^, indicating that 8-oxoG in the template reduces the binding of Gp90 D234A to DNA (Figure 6). In the presence of dATP and Mg^2+^, the misincorporation further weakens the binding affinity of Gp90 D234A to DNA.

Since the crystal structures of PaP1 DNA polymerase in complex with DNA containing 8-oxoG are not available, crystal structures of other DNA polymerases may provide insight in how Gp90 bypasses 8-oxoG. *S. solfataricus* DNA polymerase Dpo4 catalyzes 8-oxoG bypass efficiently and accurately. Crystal structures reveal the potential role of Arg332 in stabilizing the anti-conformation of 8-oxoG through the hydrogen bond or ion-dipole pair, which results in an increased enzymatic efficiency for dCTP insertion and a less favorable formation of a Hoogsteen pair between 8-oxoG and dATP [13]. DNA polymerase κ can bypass 8-oxoG in an error-prone manner by mainly inserting dATP. dATP:8-oxoG insertion events are two-fold more efficient than dCTP:G insertion events. Crystal structures of a complex of human Pol κ and DNA containing 8-oxoG show that the N-terminal extension of Pol κ stabilizes its little finger domain that surrounds the Hoogsteen base pair of 8-oxoG and incoming dATP, explaining the increase in efficiency for dATP incorporation opposite 8-oxoG [16].

## 5. Conclusions

In this work, we investigated the steady-state and pre-steady-state kinetics of nucleotide incorporation opposite G or 8-oxoG using Gp90 D234A, which has eliminated the ssDNA and dsDNA exonuclease activities. Among four dNTPs, dCTP was preferentially incorporated opposite G or 8-oxoG, exhibiting similar misincorporation frequencies of 10^−4^ to 10^−5^. Misincorporation opposite G inhibits its subsequent extension, whereas misincorporation opposite 8-oxoG does not inhibit its subsequent extension. dCTP incorporation opposite G and dCTP or dATP incorporation opposite 8-oxoG show a fast burst phase, indicating that the incorporation step is faster than the subsequent dissociation of polymerase from DNA. The burst incorporation efficiency is decreased in the following order: dCTP:G > dCTP:8-oxoG > dATP:8-oxoG. 8-oxoG in the template does not affect the binding of Gp90 D234A to DNA in the binary complex, in contrast to that in the ternary complex in the presence of dCTP and Mg^2+^. dATP misincorporation opposite 8-oxoG further weakens the binding affinity of Gp90 D234A to DNA. This study reveals that Gp90 D234A can ensure error-free bypass of 8-oxoG, providing an understanding of the DNA replication while encountering oxidation lesions for *P. aeruginosa* phage PaP1.

## Figures and Tables

**Table 1:** Oligodeoxynucleotieds used in this study.

27-mer	5′-GCTACAGAGTTATGGTGACGATACGTC-3′
28C-mer	5′-GCTACAGAGTTATGGTGACGATACGTCC-3′
28A-mer	5′-GCTACAGAGTTATGGTGACGATACGTCA-3′
30-mer	5′-TTTGCTACAGAGTTATGGTGACGATACGTC_dd_-3′
62-mer	3′-CGATGTCTCAATACCACTGCTATGCAGG*CTATCTCGCCTAATGATATGATGTAAT CTTAAGT-5′

G*: G or 8-oxoG.

**Table 2:** Steady-state kinetic analysis of single-base incorporation by Gp90 D234A.

Template Base	dNTP	*K*_m,dNTP_ μM	*k*_cat_, ×10^−3^min^−1^	*k*_cat_/*K*_m_, μM^−1^min^−1^	Misincorporation Frequency
G	C	(1.3 ± 0.1) × 10^−3^	840 ± 10	650	
	A	12 ± 1	750 ± 50	0.06	9.3 × 10^−5^
	G	7.7 ± 0.5	800 ± 10	0.10	1.5 × 10^−4^
	T	4.2 ± 0.5	430 ± 8	0.10	1.5 × 10^−4^
8-oxoG	C	2.9 ± 0.1	850 ± 10	0.85	
	A	300 ± 36	190 ± 8	6.1 × 10^−4^	7.2 × 10^−4^
	G	73 ± 9	0.15 ± 0.01	2.1 × 10^−6^	2.5 × 10^−4^
	T	120 ± 9	0.25 ± 0.01	8.3 × 10^−6^	1.0 × 10^−5^

**Table 3:** Steady-state kinetic parameters for next-base extension by Gp90 D234A.

Template Base	Primer X	*K*_m,dGTP_ μM	*k*_cat_, ×10^−2^min^−1^	*k*_cat_/*K*_m_, μM^−1^min^−1^	Efficiency relative to G:C
G	C	0.05 ± 0.01	49 ± 1	9.8	1
	A	0.04 ± 0.01	5 ± 0.2	1.3	8-fold less
	T	0.05 ± 0.01	1 ± 0.1	0.2	49-fold less
8-oxoG	C	20 ± 2	40 ± 1	2.0 × 10^−2^	490-fold less
	A	20 ± 2	46 ± 1	2.3 × 10^−2^	490-fold less
