# Erratum: Gu, S. et al. Error-Free Bypass of 7,8-dihydro-8-oxo-2′-deoxyguanosine by DNA Polymerase of *Pseudomonas aeruginosa* Phage PaP1. *Genes* 2017, *8*, 18

**DOI:** 10.3390/genes8030091

**Published:** 2017-03-03

**Authors:** Shiling Gu, Qizhen Xue, Qin Liu, Mei Xiong, Wanneng Wang, Huidong Zhang

**Affiliations:** 1 College of Pharmacy and Bioengineering, Chongqing University of Technology, No. 69 Hongguang Street, Banan District, Chongqing 400054, China; shilinggu@foxmail.com (S.G.); liuqin@2014.cqut.edu.cn (Q.L.); 2 Public Health Laboratory Sciences and Toxicology, West China School of Public Health, Sichuan University, No. 17 People’s South Road, Chengdu 610041, China; xueqzhen@163.com(Q.X.); xhmei2016@126.com (M.X.)

The authors wish to make the following correction to their paper [1]. The title of the paper should be corrected to “Error-Free Bypass of 7,8-dihydro-8-oxo-2′-deoxyguanosine by DNA Polymerase of *Pseudomonas aeruginosa* Phage PaP1”. Additionally, the affiliation 1 should be corrected to “College of Pharmacy and Bioengineering, Chongqing University of Technology, No. 69 Hongguang Street, Banan District, Chongqing 400054, China”. The authors would like to apologize for any inconvenience caused. The change does not affect the scientific results. The manuscript will be updated and the original will remain online on the article webpage.
